# Prognostic value of the HALP score in metastatic castration-resistant prostate cancer: an analysis combined with time to castration resistance

**DOI:** 10.3389/fonc.2024.1431629

**Published:** 2024-12-05

**Authors:** İvo Gökmen, Nazan Demir, Pınar Peker, Erkan Özcan, Fahri Akgül, İsmail Bayrakçı, Didem Divriklioğlu, Bülent Erdoğan, Sernaz Topaloğlu, Muhammet Bekir Hacıoğlu

**Affiliations:** ^1^ Division of Medical Oncology, Department of Internal Medicine, Trakya University School of Medicine, Edirne, Türkiye; ^2^ Department of Medical Oncology, Sultan I. Murat Public Hospital, Edirne, Türkiye; ^3^ Division of Medical Oncology, Department of Internal Medicine, Ege University School of Medicine, Izmir, Türkiye

**Keywords:** prostate cancer, castration resistance, HALP score, time to castration resistance, prognostic tools

## Abstract

**Objective:**

The aim of our study was to assess the impact of the combination of HALP score with TTCR score on OS and PFS in PC patients who developed castration resistance.

**Patients and methods:**

The study enrolled 152 patients with metastatic disease who had received either ARTAs or docetaxel as first-line treatment. The median cut-off was 30.83 months for the HALP score and 16.1 months for TTCR determined by ROC analysis. Based on these cut-off values, patients were categorized into low-high HALP score and TTCR <16.1 months-TTCR ≥16.1 months groups. The combination of HALP score and TTCR was then stratified by risk into three new groups: Factor 0, Factor 1, and Factor 2.

**Results:**

PFS was significantly shorter in the TTCR <16.1 months group compared to the TTCR ≥16.1 months group, as well as in the low-HALP score group compared to the high-HALP score group. Furthermore, as the number of factors increased, a significant increase in OS and PFS was observed in the groups formed by the combination of HALP score and TTCR.

**Conclusion:**

We have validated the predictive capability of combining low HALP score (<30.38) and short TTCR (<16.1 months) parameters in estimating the OS and PFS durations of mCRPC patients, both recognized as unfavorable prognostic indicators.

## 1 Introduction

Prostate cancer (PC) stands as the most prevalent solid organ tumor among males and ranks as the second leading cause of cancer-related mortality in this demographic (1). Upon diagnosis, approximately 78% of patients present with early-stage disease confined to the prostate capsule, while 12% exhibit regional lymph node metastases and 6% showcase distant metastases. Nevertheless, a majority of patients progress to develop distant metastases over time (2).

Current guidelines now state that monotherapy with androgen deprivation therapy (ADT) is no longer the standard initial treatment for hormone-sensitive prostate cancer (mHSPC) and should not be favored (3, 4). However, in countries where the use of androgen receptor-targeting agents (ARTAs) is restricted due to reimbursement limitations by public health insurance systems, many patients still receive ADT monotherapy. Although initial response rates to ADT range from 80% to 90%, treatment response is often transient, and cancer progression occurs in a significant number of patients within 6 months to several years, leading to metastatic castration-resistant prostate cancer (mCRPC), which is fatal (5, 6). In this population, docetaxel or ARTAs (abiraterone acetate or enzalutamide) may be considered as first-line treatment alongside ADT. Estimated survival ranges from 13.2 to 23.2 months, although some patients may respond favorably to these treatments (7).

mCRPC is a heterogeneous disease with variable characteristics and prognosis, representing a large patient population. The classification of these patients relies on common indicators such as prostate-specific antigen (PSA), alkaline phosphatase (ALP), lactate dehydrogenase (LDH), Eastern Cooperative Oncology Group erformance status (ECOG PS), Gleason score (GS), and tumor size (T), nodal involvement (N), and metastasis (M) stage (TNM stage) (8, 9). Unfortunately, these indicators have limited prognostic accuracy. Clinicians still require novel prognostic patterns and biomarkers to aid in the classification and management of these patients. Some prognostic indices based on inflammation and/or nutritional scores, such as the neutrophil-lymphocyte ratio (NLR) (10), platelet-lymphocyte ratio (PLR) (11), C-reactive protein/albumin ratio (CAR) (12), hemoglobin (HGB) level (13), and prognostic nutritional index (PNI) (14), have previously been utilized to predict the prognosis of patients with mCRPC who develop castration resistance following ADT treatment. Combinations of these parameters are known to provide better predictions of disease prognosis compared to single indices.

In this context, the HALP score—based on HGB, albumin, lymphocyte, and platelet levels—has recently emerged as a marker that evaluates both immune and nutritional status. Many studies have reported the HALP score’s utility as a reliable prognostic index in various types of cancer (15–22). However, data on PC patients are limited (23, 24), and to our knowledge, no study has yet evaluated the HALP score in mCRPC patients.

Given the importance of identifying reliable prognostic markers in prostate cancer, particularly in advanced stages, research has shown that a shorter time to castration resistance (TTCR) is linked to poorer overall survival (OS) in PC patients, both after diagnosis and after developing castration resistance (25–27). Wenzel et al. (27) suggested that the duration of treatment response before castration resistance is influenced by both patient and tumor characteristics. They further hypothesized that genetic mutations or alterations in the host or tumor may also contribute.

A previous study has shown that the combination of the CAR and TTCR can be used as a prognostic marker in mCRPC patients (12). In our study, we investigated the prognostic value of the HALP score and TTCR in mCRPC patients who developed resistance after ADT treatment. We aimed to determine whether their combination could be utilized to predict prognosis more accurately than current methods.

## 2 Materials and methods

### 2.1 Data collection, assessments, and follow-up

We adhered to the ethical standards of institutional and national research committees, as well as the 1964 Declaration of Helsinki and its subsequent revisions. The method and procedure of the study were approved by the Ethics Committee of Trakya University Faculty of Medicine (protocol code TÜTF-GOBAEK 2023/362) on October 9, 2023.

Patients histopathologically diagnosed with prostate adenocarcinoma in our medical oncology department between January 2010 and September 2023, who had previously failed ADT, were retrospectively reviewed. A total of 152 patients were enrolled, receiving docetaxel or ARTAs such as their primary treatment, with metastatic disease at the time of castration resistance development. Treatment decisions were made based on disease progression, toxicity, patient rejection of treatment, and physician preference. PSA levels at diagnosis of GS, mHSPC, and mCRPC, along with age before initial treatment for mCRPC, ECOG PS, and patient demographics, were recorded. In mCRPC patients, laboratory parameters including HGB, lymphocyte count, platelet count, serum albumin, ALP, and LDH levels were recorded if obtained within 2 weeks before the initiation of treatment. Lymphocyte and platelet counts, as well as serum HGB and albumin levels, were retrieved from the laboratory information system to calculate the HALP score. The HALP score was calculated using the following formula: [HGB (g/L) × albumin (g/L) × lymphocytes (/L)]/platelets (/L). Patients with factors that could potentially affect inflammatory parameters (e.g., active infection, inadequate organ function, or those receiving anti-inflammatory treatment) were excluded from the analysis.

The clinical stage was determined using standardized TNM criteria based on digital rectal examination, computed tomography, magnetic resonance imaging, and bone scintigraphy. Risk stratification was based on the criteria utilized in the CHAARTED and LATITUDE trials at the time of mCRPC diagnosis. Patients demonstrating evidence of disease progression (e.g., increased PSA levels, new metastases, or progression of existing metastases) with castrate serum testosterone levels (<50 ng/dl or <1.7 nmol/l) were classified as having developed mCRPC. TTCR was defined as the duration from the initiation of ADT treatment in mHSPC patients to the first reported date of mCRPC. This duration reflects the time it takes for the disease to develop resistance to ADT therapy. Regarding PSA progression, three consecutive increases of 50% or more in nadir PSA (2.0 ng/ml) were considered as a reference. Disease progression was determined by an increase in the number or size of lesions assessable according to the Response Evaluation Criteria in Solid Tumors 1.1 (RECIST 1.1) or the presence of new lesions on imaging studies.

The primary and secondary endpoints of the study were OS and progression-free survival (PFS) after the development of mCRPC, respectively. OS was defined as the duration from the diagnosis of mCRPC until the patient’s death or the final visit. PFS was calculated from the initiation of the first treatment for mCRPC until the last day of the study or evidence of progressive disease.

### 2.2 Statistical analyses

All analyses were conducted using the IBM (International Business Machines) SPSS (Statistical Package for the Social Sciences) Statistics for Windows, Version 27.0 (Armonk, N.Y., USA) and MedCalc Statistical Software Ltd. (Ostend, Belgium; https://www.medcalc.org; 2020). Receiver operating characteristic (ROC) curve analysis was utilized to plot the curves for the HALP score and TTCR. Subsequently, sensitivity and specificity values were transferred to Microsoft Excel, where the cut-off value was determined as the point at which the sensitivity and specificity values reached the highest total score. The cohort was stratified into three groups based on HALP score and TTCR, and differences between them were analyzed using ANOVA or chi-square tests. The Kruskal-Wallis test was employed to assess continuous variables without a normal distribution across groups. Survival curves were constructed using the Kaplan-Meier method, and differences were analyzed using the log-rank test. Univariate and multivariate analyses for OS were performed using the Cox proportional hazards regression model. All tests were two-sided, with differences considered statistically significant if the p-value was less than 0.05.

## 3 Results

### 3.1 Patient characteristics

In our study, the median age of the 152 patients was 70 years (range: 53-82 years). Among them, 52 patients (34.2%) had a pre-treatment ECOG PS of 0, while the remaining 100 patients (65.8%) had a ECOG PS of 1 or 2. The mean pre-treatment levels of HGB, albumin, lymphocytes, and platelets were 12.8 g/dL, 4.4 g/dL, 1.4 × 10^3/μL, and 225 × 10^3/mm^3, respectively, with a mean HALP score of 30.83 (range: 1.95-82.24). Among the patients, 109 (71.1%) met the CHAARTED criteria for high-volume disease, while 92 (60.5%) met the LATITUDE criteria for high-risk disease. Distant metastases were most commonly observed in the bone (n=123, 80.9%), with visceral metastases detected in 49 patients (32.2%). The median TTCR was 14.4 months (range: 4.3-53.7 months). Docetaxel was administered as the first-line treatment for mCRPC in 94 patients (61.8%), while ARTAs were used in 58 patients (38.2%). Throughout the observation period of the study, 146 patients (96.1%) received ARTAs, and 140 patients (92.1%) underwent docetaxel therapy (see **Table 1**).

**Table 1:** Clinicopathological characteristics and treatment data of 152 patients diagnosed with mCRPC.

Patient characteristics	N, (%)
Age at the time of mCRPC, years. *Median, SD, range*	70 ± 7.5 (53-87)
ECOG PS, 0 ≥1	52 (34.2%) 100 (65.8%)
Serum PSA level at the time of mHSPC, ng/ml	62.4 (12.8-654.2)
Serum markers at the diagnosis of mCRPC. *Median, range*	
PSA level, ng/dl Hemoglobin, g/dl Lymphocyte, 10^3/μL Platelets, 10^3/mm^3 Lactate dehydrogenase, U/L Alkaline phosphatase, U/L Albumin, g/dl HALP score	12.8 (2.6-64.8) 12.6 (8.1-18.8) 1.40 (0.16-3.96) 225 (102-498) 208 (100-812) 183 (48-1497) 4.4 (2.4-5.1) 30.83 (1.95-92.24)
Clinical T stage, ≤3 4	136 (89.5%) 16 (10.5%)
Gleason score, ≤8 ≥9	80 (52.6%) 72 (47.4%)
(CHAARTED), Low High	43 (28.3%) 109 (71.1%)
(LATITUTE), Low High	60 (39.5%) 92 (60.5%)
Locoregional LN metastasis at the time of mCRPC (present)	93 (61.2%)
Distant metastasis at the time of mCRPC	
Bone (Present) Bone (≥4) Visceral metastasis (lung, liver, others)	123 (80.9%) 95 (62.5%) 49 (32.2%)
Time to the development of castration resistance, months. *Median, range*	14.6 (4.3-53.7)
First line treatment for mCRPC	
Docetaxel ARTAs	94 (61.8%) 58 (38.2%)
ARTAs during the treatment process	88 (57.9%)
Docetaxel administration during the treatment process	46 (30.3%)

mCRPC, metastatic castration-resistant prostate cancer; mHSPC, metastatic hormone-sensitive prostate cancer; ECOG PS, Eastern Cooperative-Oncology Group Performance Status; PSA, prostate-specific antigen; ARTAs, androgen receptor-targeting agents.

### 3.2 Evaluation of the HALP score and TTCR as prognostic factors

**Figure 1:**
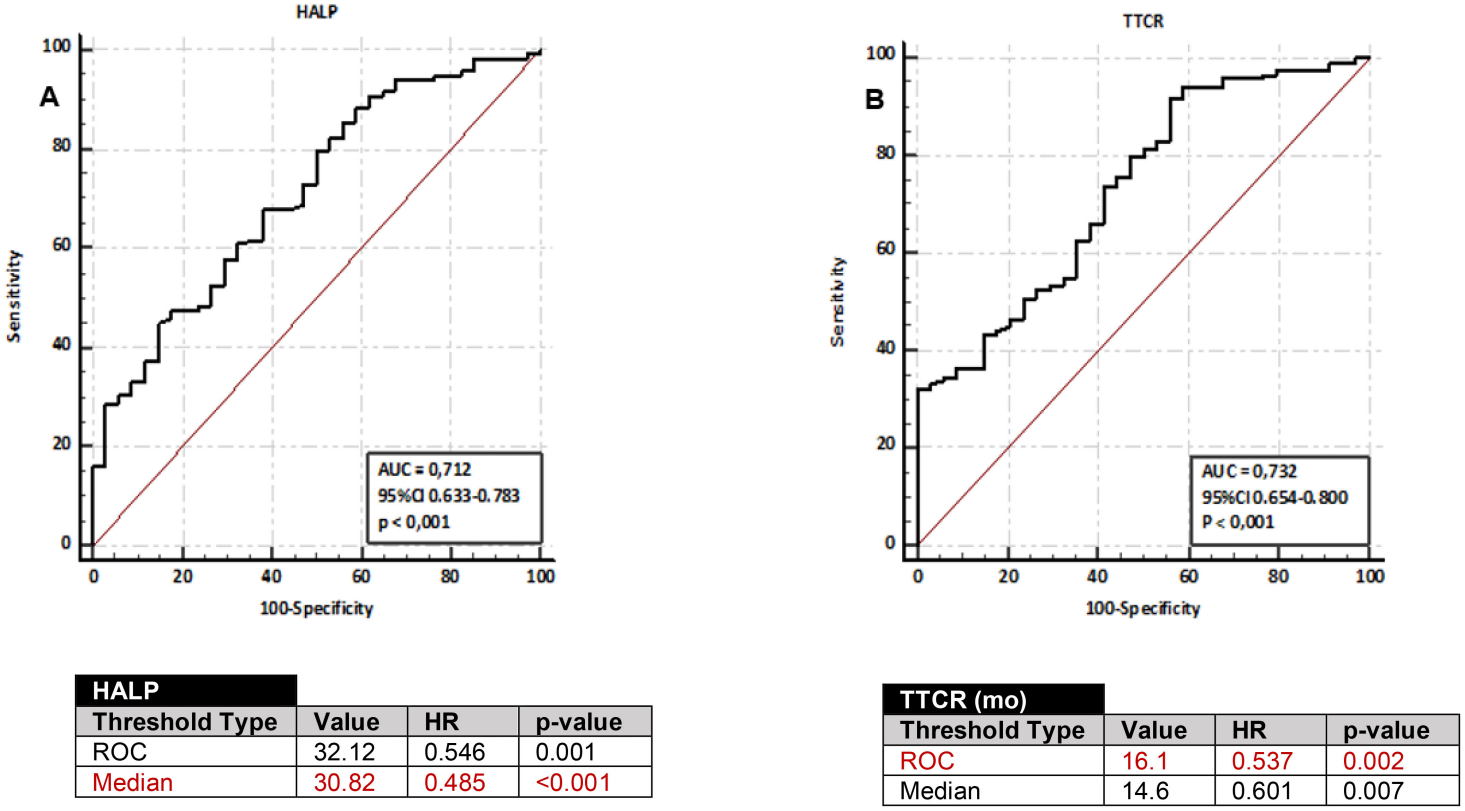
ROC curves for predicting overall survival in mCRPC patients using HALP score **(A)** and TTCR **(B)**. Cut-off values derived from ROC analysis and median-based cut-offs, along with performance metrics (HR and p-values) for OS, obtained through Cox regression analysis, are displayed in the accompanying tables.

We examined the optimal cut-off points for both the HALP score and TTCR in predicting mortality in mCRPC. The optimal cut-off point for mortality prediction, as determined by ROC curve analysis, was ≥32.13 months for the HALP score (area under the curve 0.712, sensitivity 66.18%, and specificity 61.77%) and ≥16.1 months for TTCR (area under the curve 0.732, sensitivity 62.71%, and specificity 61.77%). When comparing these values with the cut-off points determined by the median using Cox analysis, the results indicated that the hazard ratio (HR) was superior to the median cut-off for TTCR but not for the HALP score (see **Figure 1**).

**Figure 2:**
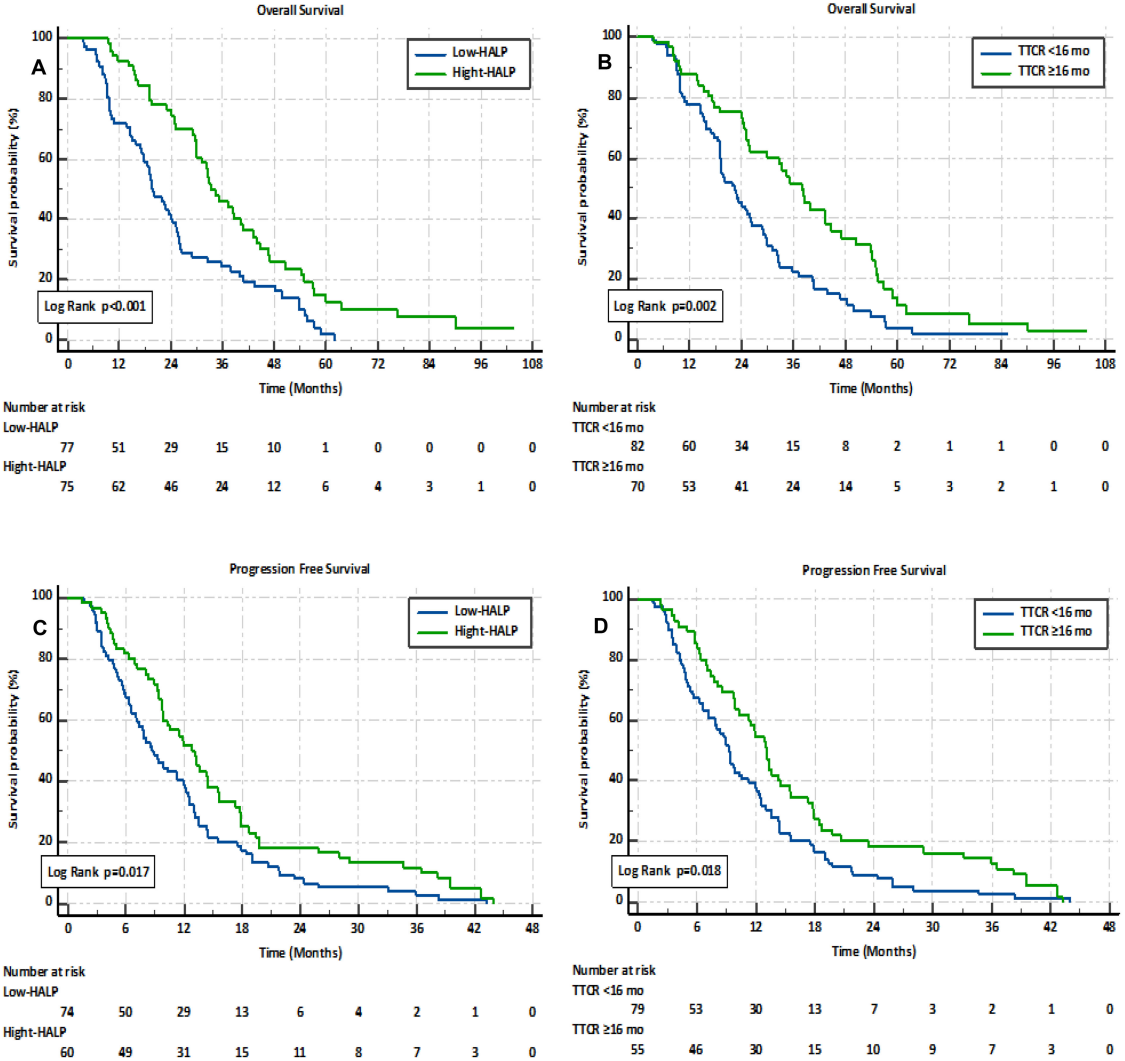
Kaplan–Meier analysis of overall survival and progression-free survival in mCRPC patients, stratified by HALP score **(A, C)** and TTCR **(B, D)**.

Based on this data analysis, the median cut-off value was utilized for the HALP score. Patients were categorized into two groups: a low HALP score group (<30.83, n=77) and a high HALP score group (≥30.83, n=75). The cut-off value from the ROC analysis was applied for TTCR, and patients were categorized into two groups: TTCR≥16.1 months (n=70) and TTCR<16 months (n=82). Kaplan-Meier analysis revealed that the low HALP score group had significantly inferior OS compared to the high HALP score group (median 19.8 months (95%CI 16.1-23.4) *vs*. 33.3 months (95%CI 27.9-38.7), *p<0.001*). Similarly, the TTCR<16.1 months group exhibited worse OS compared to the TTCR≥16.1 months group (median 23.5 months (95%CI 19.2-26.8) *vs*. 36.8 months (95%CI 30.1-43.6), *p=0.002*). Moreover, PFS was notably shorter in the low HALP score group compared to the high HALP score group and in the TTCR<16.1 months group compared to the TTCR≥16 months group (*p=0.017*, *p=0.018*, respectively) (see **Figure 2**).

Univariate and multivariate Cox analyses were conducted to further explore the prognostic significance of the HALP score and TTCR. The univariate analysis revealed that both the HALP score (HR 0.485, 95% CI 0.334-0.706, *p<0.001*) and TTCR (HR 0.537, 95% CI 0.369-0.784, *p=0.002*) were significantly associated with OS. Similarly, factors such as age ≥75 years, ECOG PS ≥1, HGB ≥12.8 g/dl, lymphocyte count ≥1.40 x 10^3/μL, high-volume disease, high-risk disease, bone metastases, and visceral metastases were identified as potential prognostic factors significantly correlated with OS. Although not statistically significant, albumin ≥4.1 g/dl and ALP ≥183 U/L were considered potential prognostic factors for OS (see **Table 2**). Additionally, both the HALP score (HR 0.499, 95% CI 0.338-0.737, *p=0.001*) and TTCR (HR 0.643, 95% CI 0.436-0.948, *p=0.026*) consistently demonstrated independent prognostic value for OS in multivariate analysis, controlling for uncorrelated prognostic factors including age, ECOG PS, ALP, high-risk disease, HALP score, and TTCR.

**Table 2:** Univariate Cox proportional hazards analysis of factors affecting overall survival in patients with mCRPC.

Covariates	HR	95%CL	p-value
Age at the time of mCRPC (≥70)	1,178	0,819-1,695	0,377
ECOG PS, (≥1)	1,675	1,120-2,504	**0,012**
Serum PSA level at the time of mHSPC, ng/ml (≥62.4 ng/ml)	0,855	0,595-1,231	0.4
Serum markers at the time of mCRPC			
PSA level, (≥12.8 ng/ml) Hemoglobin, (≥12.6 g/dl) Lymphocyte, (≥1.40 10^3/μL) Platelets, (≥ 225 10^3/mm^3) Lactate dehydrogenase, (≥208 U/L) Alkaline phosphatase, (≥183 U/L) Albumin, (≥4.1 g/dl) HALP, (≥30.83)	1,193 0,591 0,609 0,909 0,967 1,348 0,682 0,485	0,815-1,745 0,409-0,855 0,421-0,880 0,621-1,331 0,689-1,460 0,960-1,997 0,474-0,983 0,334-0,706	0,363 **0,006** **0.01** 0,625 0,967 0,082 **0.04** **<0.001**
Clinical T stage, (>4)	0,809	0,465-1,405	0,451
Gleason score, (≥9)	1,236	0,861-1,774	0,251
(CHAARTED), (High)	1,543	1,030-2,313	**0,036**
(LATITUTE), (High)	1,559	1,065-2,282	**0,022**
Locoregional LN metastasis at diagnosis of mCRPC (present)	0,872	0,606-1,255	0,461
Distant metastasis at diagnosis of mCRPC			
Bone metastasis (Present) Visceral metastasis (present) (lung, liver, etc.)	1,761 1,552	1,080-2,871 1,015-2,282	**0,024** **0,042**
Time to the development of castration resistance, (≥ 16 months)	0,537	0369-0,784	**0,002**
First line treatment at diagnosis of mCRPC			
Docetaxel ARTAs	1,064	0,702-1,613	0,771
ARTAs administration during the treatment process	0,850	0,569-1,271	0,430
Docetaxel administration during the treatment process	0,796	0,537-1,182	0,259

Bold values indicate statistically significant p-values (p < 0.05).mCRPC, metastatic castration-resistant prostate cancer; mHSPC, metastatic hormone-sensitive prostate cancer; ECOG PS, Eastern Cooperative-Oncology Group Performance Status Scale; PSA, prostate-specific antigen; ARTAs, androgen receptor-targeting agents.

### 3.3 Evaluation of the combination between the HALP score and TTCR

**Figure 3:**
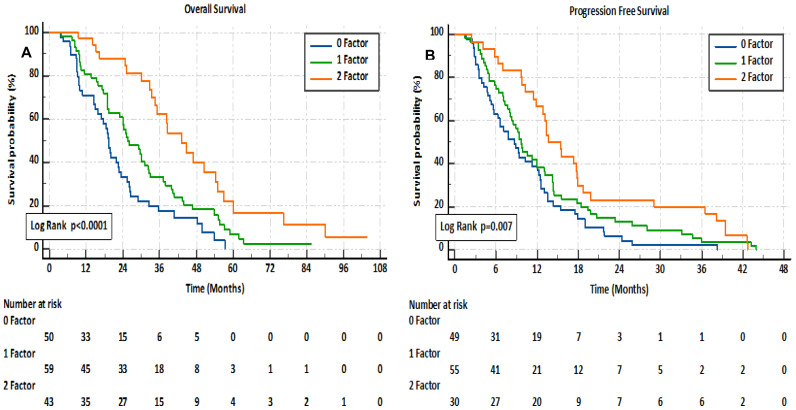
Kaplan–Meier analysis of overall survival **(A)** and progression-free survival **(B)** in mCRPC patients, based on a combination of HALP score and TTCR.

We assessed whether combining the HALP score and TTCR could enhance accuracy in predicting patient prognosis. The study population was divided into three groups based on this new combination. The factor 0 “0F” group comprised patients with a low HALP score and TTCR<16.1 months; the factor 1 “1F” group included those with a high HALP score and TTCR<16.1 months, or a low HALP score and TTCR≥16.1 months; and the factor 2 “2F” group consisted of patients with a high HALP score and TTCR≥16.1 months. By combining the HALP score and TTCR, there was a significant gradual increase in both OS (0F: median 19.5 months (95% CI 17.8-21); 1F: 25.4 (95% CI 19.2-31.7); 2F: 43.2 (95% CI 33.7-52.6); *p<0.001*) and PFS (*p=0.007*) as the number of factors increased (see **Figure 3**). Significant differences were observed between the groups in various blood markers, including HGB, lymphocytes, platelets, and albumin. The presence of bone metastases, visceral metastases, high-risk disease, and an ECOG PS of 0 were also found to have an inverse correlation with the number of available factors (see **Table 3**).

**Table 3:** Clinicopathologic characteristics of mCRPC patients stratified into three groups based on HALP score and TTCR.

Patient characteristics	0F	1F	2F	p-value
	N,%	N,%	N,%	
Age at the time of mCRPC	68 (53-84)	71 (54-87)	70 (57-83)	0,847
ECOG PS, (≥1)	36 (72.0)	41 (69.5)	23 (53.5)	0,067
Serum PSA level at the time of mHSPC, ng/ml	53.8 (12-504)	62.0 (13-654)	68 (12-541)	0,687
Serum markers at the time of mCRPC				
PSA level, (ng/ml) Hemoglobin, (g/dl) Lymphocyte, (10^3/μL) Platelets, (10^3/mm^3) Lactate dehydrogenase, (U/L) Alkaline phosphatase, (U/L) Albumin, (g/dl) HALP score	15.6 (2.3-43.6) 11.7 (8.2-18-8) 1.21 (0.16-2.13) 254 (148-498) 205 (101-812) 202 (55-1497) 3.9 (2.3-5.0) 20.64 (1,95-30.69)	13.4 (2.6-64.8) 12.4 (8.1-14.6) 1.40 (0.60-3.96) 208 (148-486) 211 (100-642) 201 (43-522) 4.0 (2.6-4.7) 31.71 (8,57-92.24)	12.0 (3.0-43.9) 13.5 (9.9-18.2) 1.93 (0.82-3.58) 215 (139-388) 201 (101-812) 101 (41-542) 4.2 (3.3-5.1) 46.52 (31.49-92.18)	0,064 **<0.001** **<0.001** **0,035** 0,305 **0,014** **<0.001**
Clinical T stage, ≤3 4	4 (92.0) 4 (8.0)	52 (88.1%) 7 (11.9%)	38 (88.4%) 5 (11.6%)	0,776
Gleason score, ≤8 ≥9				
(CHAARTED), Low High	10 (20.0) 40 (80.0)	15 (25.4%) 44 (74.6%)	18 (41.9%) 25 (58.1%)	0,054
(LATITUTE), Low High	12 (24.0) 38 (76.0)	22 (37.3%) 37 (62.7%)	26 (60.5%) 17 (39.5%)	**0,001**
locoregional LN metastasis at diagnosis of mCRPC (present)	26 (52.0)	40 (67.8%)	27 (62.8%)	0,234
Distant metastasis at diagnosis of mCRPC				
Bone (Present) Visceral metastasis (lung, liver, others)	46 (92.0) 20 (40.0)	46 (78.0%) 22 (37.3%)	31 (71.0%) 7 (16.%)	**0,039** **0,029**
Time to development of castration resistance				
≥16 months <16 months	0 (0) 50 (100.0)	32 (54.2) 27 (45.8)	59 (100.0) 0 (0)	**<0.001**
First line treatment at diagnosis of mCRPC				
Chemotherapy ARTAs	35 (70.0%) 15 (30.0%)	38 (64.4%) 21 (35.6%)	21 (48.8%) 22 (51.2%)	0,097
Administration of ARTAs during the treatment process (present)	29 (58.0%)	37 (62.7%)	22 (51.2%)	0,506
Docetaxel administration during the treatment process (present)	19 (38.0%)	18 (30.5%)	9 (20.9%)	0,202

Bold values indicate statistically significant p-values (p < 0.05).mCRPC, metastatic castration-resistant prostate cancer; mHSPC, metastatic hormone-sensitive prostate cancer; ECOG PS, Eastern Cooperative-Oncology Group Performance Status Scale; PSA, prostate-specific antigen; ARTAs, androgen receptor-targeting agents.

## 4 Discussion

In our study, we aimed to investigate the prognostic value of pre-treatment HALP score, TTCR, and their combination in predicting both OS and PFS in patients diagnosed with mCRPC who developed resistance following ADT and were subsequently treated with ARTAs or docetaxel as first-line therapy. This retrospective analysis focused on patients treated at our clinic after developing castration resistance. The key findings are detailed below.

Recent advances in the treatment of mCRPC with new therapeutic options have underscored the increased need for predictive and prognostic markers to inform individualized treatment selection. In recent years, there has been a growing number of studies focusing on the roles of inflammatory response and nutritional status in predicting prognosis in mCRPC (10, 11, 14, 28, 29). These factors may reflect the onset and progression of cancer, indirectly forecast response to anti-tumor therapy, and determine survival duration in clinical practice (30). Serum albumin, a negative acute-phase marker synthesized in the liver, serves as an indicator of nutritional status. Hypoalbuminemia, which frequently occurs in cancer patients, may be linked to compromised oral intake and catabolic processes, as well as systemic inflammation and heightened cytokine release. Consequently, this may weaken the immune response against cancer cells (31). Numerous studies have highlighted that hypoalbuminemia in mCRPC is associated with shorter survival outcomes (32, 33).

Lymphocytes play a crucial role in the immune system. They can secrete cytokines such as interferon-γ and tumor necrosis factor-alpha (TNF-α), which may enhance prognosis by inducing apoptosis and suppressing cancer cell proliferation, invasion, and migration (34, 35). In summary, lymphocytopenia may contribute to tumor growth. Several studies have demonstrated a direct relationship between HGB levels and both survival and tumor development in cancer patients (36, 37). Platelets are pivotal in the metastatic potential of tumor cells, with tumor cell-induced platelet aggregation (TCIPA) being associated with high metastatic potential across various cancer types. Additionally, platelets may shield tumor cells from immune attack. They can also facilitate tumor angiogenesis through vascular endothelial growth factor (VEGF) and other inflammatory mediators, while inhibiting tumor necrosis via TNF-α (38, 39).

In light of these data, the HALP score, derived from HGB, albumin, lymphocyte, and platelet values, may be regarded as a comprehensive index measuring the nutritional status and immune health of patients. In 2015, Chen et al. initially reported that a higher pre-operative HALP score (cut-off value of 58.8) was associated with improved survival outcomes in gastric cancer patients (15). Shortly thereafter, Jiang et al. identified a higher mortality risk in locally advanced colorectal cancer patients with a low pre-operative HALP score (cut-off value of 26.5) (16). Subsequent studies have confirmed the HALP score as a useful prognostic tool for predicting the prognosis of patients with various cancer types, including pancreatic and biliary tract cancer (17, 18), lung cancer (19, 20), and urologic cancers such as renal cell carcinoma (21, 22). To date, the HALP score has been investigated in two distinct studies within the context of PC, yielding differing results. In a 2019 study by Guo et al. the pre-operative HALP score was significantly correlated with PFS in both metastatic and oligometastatic PC subgroups in patients undergoing cytoreductive radical prostatectomy for PC (23). Subsequently, a study conducted by Kaya et al. in 2020 explored the pre-operative diagnostic significance of the HALP score in patients with PC but found no correlation (24). To our knowledge, there have been no previously reported studies on the prognostic significance of the HALP score in patients with mCRPC.

Nearly all previous studies have utilized X-tile or ROC curves to determine the cut-off value of the HALP score, which predominantly fell between 15.5 and 56.8 (40). There is no evidence indicating a correlation between the HALP score cut-off and tumor type. A single, fixed value may not represent the optimal threshold across all tumor types. In our study, the median cut-off value (30.83) was employed for the HALP score. Kaplan-Meier survival analysis confirmed a significant disparity in OS between the high and low HALP score groups. Patients with a high pre-treatment HALP score (≥30.83) exhibited a median OS of 33.3 months, whereas those with a low HALP score (<30.83) experienced a notably shorter OS of 19.8 months (*p<0.001*). This significance was also evident for PFS. The HALP score maintained its independent prognostic value for OS in both univariate analysis (HR 0.485, 95% CI 0.334-0.706, *p<0.001*) and multivariate analysis encompassing various patient and tumor factors (HR 0.499, 95% CI 0.338-0.737, *p=0.001*). These findings highlight that improvements in the HALP score could greatly improve survival outcomes in mCRPC patients.

The cut-off value for TTCR from the ROC analysis (16.1 months) was used in our study. TTCR was also confirmed to be an independent predictor for OS in mCRPC in both univariate and multivariate analyses. Patients with TTCR ≥16.1 months had a mean OS of 36.8 months, whereas those with TTCR <16.1 months had a significantly shorter OS (23.3 months). This trend was also observed for PFS. Many studies have used a 12-month cut-off as the optimal threshold for TTCR in patients treated with ADT during the mHSPC period who subsequently developed castration resistance. Miyake et al. in their study stratifying TTCR, found that those with a TTCR of ≤6 months had the worst prognosis (25, 26, 41). Additionally, it has been demonstrated that short TTCR durations were strongly associated with poor prognosis following the development of mCRPC in patients undergoing combination therapy (ARTAs, docetaxel) with ADT during the mHSPC period (27).

Finally, this study confirmed that it is possible to predict both OS and PFS durations more accurately by categorizing mCRPC patients into three groups based on parameters defined as poor prognostic factors: low HALP score (<30.38) and short TTCR (<16.1 months). In the newly established prognostic model, a gradual increase in survival was observed for both OS (0F: median 19.5 months; 1F: 25.4 months; 2F: 43.2 months; *p<0.001*) and PFS (*p=0.007*) as the number of factors increased. Harrel’s 0.701 c-index value can be indicative of the combined score’s superior performance in distinguishing patients within the best prognostic group compared to the individual use of HALP score (c-index 0.641) and TTCR (c-index 0.639). These results are not surprising, as the combination of these factors incorporates variations in various host and tumor-related poor prognostic factors such as low ECOG PS, anemia, lymphopenia, hypoalbuminemia, high-risk disease, and metastasis.

This study has several limitations. Firstly, although it is the first study to demonstrate both the HALP score and the HALP score in combination with TTCR for OS as independent prognostic factors in patients with mCRPC, its retrospective design spanning 13 years and the potential for bias in patient selection reduce the study’s reliability. Secondly, the evaluation of patients treated at a single center and the small number of patients are limitations. The small sample size and the HALP score and TTCR cut-off values used may not be sufficient to reflect prognosis in other cohorts. Additionally, the results cannot be generalized to all mCRPC patients, as we did not focus on patients receiving combination therapy during a castration-sensitive phase. Finally, the failure to evaluate other potential prognostic markers such as NLR (10) and PLR (11), and PNI (14) is another limitation.

## 5 Conclusion

In conclusion, the HALP score emerges as a reliable, simple, easily accessible, and cost-effective index for predicting prognosis in mCRPC patients. A low HALP score may serve as a clinical indicator for implementing nutritional support in mCRPC patients. Moreover, the combined utilization of the HALP score and TTCR may enhance the accuracy of prognosis prediction in mCRPC patients. This approach is believed to effectively identify patients who could benefit from therapy. Multicenter studies involving larger cohorts are warranted to provide a more conclusive assessment.

## Data Availability

The original contributions presented in the study are included in the article/supplementary material. Further inquiries can be directed to the corresponding author.
